# The Family Physician

**Published:** 1994

**Authors:** Kirsten L. Barry, Michael F. Fleming

**Affiliations:** Kristen L. Barry, Ph.D., is an associate scientist and Michael F. Fleming, M.D., M.P.H., is an associate professor in the Department of Family Medicine, University of Wisconsin, Madison, Wisconsin

## Abstract

Because they often provide care for every member of a family, family physicians have the opportunity to observe and treat the repercussions of one person’s alcohol problem on all family members. Recent trends in diagnosis and treatment of alcohol-related disorders have encouraged greater involvement by family physicians in the care of a patient with alcohol problems.

Alcohol-related disorders are among the most common medical disorders family physicians encounter in their practices (Brown 1992). Family physicians have developed many of the skills practiced by internists, obstetricians, psychiatrists, pediatricians, and geriatricians and possess a breadth of expertise that gives them special abilities to detect and treat the alcohol and other drug problems they see in their practices. In addition, because these physicians often work with entire families, they are in a unique position to observe relationships among the family members and the effect that one person’s alcoholism has on the family. The following case illustrates some of these opportunities.

A family of four, after relocating in south central Wisconsin, chose a family physician as their primary care physician from a list of physicians provided by their health maintenance organization.

The first family member to seek medical attention from the family physician was the 8-year-old boy, Paul, who had chronic headaches. A neurological examination was completed and revealed no structural lesion or other medical problems. His headaches continued, despite reassurances by the physician that there was no apparent medical reason for them.

The second family member to seek medical attention was Paul’s older sister, Mary, who was 16 years old and pregnant. During a prenatal examination that the family physician conducted, Mary revealed a history of sexual abuse as a child. The physician also noted that Mary was depressed and suicidal.

The third family member to seek medical attention was Mary and Paul’s mother, Margaret, who wanted something for her nerves. She was having difficulty sleeping. On further questioning, Margaret admitted to daily alcohol use and a family history of alcoholism.

The last family member to seek medical attention from the family physician was Mary and Paul’s father, John. He wanted a refill of his “stomach medication” that he had received from his previous physician. During the routine examination, the family physician also noted that John had hypertension and a history of alcohol withdrawal.

Each of the family members was affected in some way by the alcohol abuse in the family. The family physician first became suspicious that the parents could have a problem with alcohol during the daughter’s prenatal visit. Her suspicion was confirmed with the appearance of the mother. If the family practitioner had seen only one family member, as often occurs in a specialist’s office, she would have been less likely to identify the primary issue affecting this family. It is easy to focus on an individual with a medical problem such as headaches, adolescent pregnancy, sleeping problems, or chronic abdominal pain and to overlook the relationship between these problems and an alcohol problem in the family.

A further strength inherent in the role of the family physician is the opportunity to develop a trusting, supportive relationship with all the family members. Caring for patients over a long period of time allows family physicians more occasions to discuss sensitive issues and increases the likelihood of their being able to help patients change behaviors such as destructive alcohol use.

Family physicians receive extensive training in public health issues of medical care during their residency training programs, which is essential for the primary and secondary prevention of alcohol problems. Primary prevention involves averting problems; secondary prevention involves early intervention before problems become unmanageable. The public health issues taught to family physicians are applied across diseases and include clinical experiences in community settings, screening asymptomatic individuals, and using brief and simple intervention techniques. The methods family physicians learn minimize the use of costly high-tech diagnostic and treatment methods, which helps to reduce overall health care costs while providing patients with earlier, more appropriate, and less invasive care options.

Training family physicians in the public health model corresponds to recent shifts in theories of prevention and treatment of alcohol-related problems. For instance, we now recognize that:

- The majority of people experiencing health problems secondary to their alcohol and other drug use are at-risk users rather than alcoholics and addicts (Fleming and Barry 1992).
- There is a direct correlation between levels of alcohol consumption and health effects. Adverse effects begin to occur at 12 drinks per week for women and 15 drinks per week for men. Binge drinking (five or more drinks per occasion) also is correlated with health effects, in particular, with traumatic injuries (Anderson et al. 1993; Sanchez-Craig et al. in press; National Institute on Alcohol Abuse and Alcoholism [NIAAA] 1992).
- Primary care clinicians can identify and effectively treat the majority of persons and families adversely affected by alcohol and other drug disorders (Saunders et al. 1993).
- Several specialized treatment methods in addition to the traditional 12-step treatment programs can help people change their use of alcohol and other drugs (Hester and Miller 1989).

This article will focus on these shifts in thinking and their effect on the family physician’s ability to diagnose disorders related to and care for persons adversely affected by alcohol and other drug use.

## Prevalence of Alcohol Disorders in Family Medicine

Alcohol disorders include alcohol dependence and abuse and at-risk use. Alcoholism, dependence, and abuse are defined using the American Psychiatric Association’s *Diagnostic and Statistical Manual of Mental Disorders, Fourth Edition* (DSM–IV; 1994) criteria for alcohol abuse and dependence. At-risk use is defined as men who drink 15 or more drinks per week, women who drink 12 or more drinks per week, or those of either gender who binge drink. The prevalence of a current diagnosis of alcoholism in primary care settings is 5 to 8 percent for men and 2 to 3 percent for women (Fleming and Barry 1992). Preliminary findings from a survey of 17,298 adults taken by physicians in southern Wisconsin, however, indicate that the prevalence of at-risk drinking is 15 to 20 percent for men and 8 to 10 percent for women. According to the preliminary data, approximately one-fifth of men drink 15 or more drinks per week, and 1 in 8 drink 3 or more drinks per day. By comparison, women engage in at-risk drinking less often than men.

## Recognition of At-Risk Drinkers

At-risk drinkers have been recognized as having the majority of alcohol-related problems; this has led to two changes in the diagnosis and clinical care of people who seek health care from family physicians. First, detecting at-risk and harmful drinkers is at least as important as recognizing patients who are alcohol dependent. At-risk drinkers also make up the largest percentage of drinkers with problems in primary care practices. Intervention at this stage has the potential to circumvent more severe social, medical, and psychological problems.

Indeed, epidemiologic research has established a direct relationship between alcohol use and alcohol-related problems. Put simply, the more a person drinks, the greater the number of problems he or she will have (Anderson et al. 1993; NIAAA 1992; Sanchez-Craig et al. in press). This pattern is true for most alcohol-induced health problems such as stroke, trauma, depression, heart disease, hypertension, mental status changes, alcohol-related birth defects, and alcoholic liver disease (Anderson et al. 1993; NIAAA 1992; Sanchez-Craig et al. in press). Table 1 lists the warning signs of alcohol problems commonly seen in primary care settings.

In light of the relationship between alcohol use and alcohol-related problems, a second change in diagnosis and clinical care has been the realization that questions about consumption are useful screening questions and provide a method to categorize respondents into levels of risk for alcohol-related problems. The data are similar to data obtained for hypertension or hypercholesterolemia (excess of cholesterol in the blood), in which specific cutoff points of cholesterol levels are used to assess level of risk. For example, a white middle-age man with a cholesterol level above 280 mg percent has a twofold risk of developing cardiovascular disease, compared with a man with a cholesterol level below 200 mg percent.

The traditional assumption that all patients who drink tend to underreport their alcohol use is not supported by research (Babor et al. 1989). People who are not alcohol dependent or intoxicated during screening often provide accurate information. Methods that are known to increase the accuracy of self-reporting include asking specific questions about use in the recent past, using a direct non-judgmental approach, embedding the alcohol-use questions in the context of other health behaviors such as smoking and exercise, and paying attention to nonverbal cues that suggest that the patient is minimizing his or her alcohol use. Some nonverbal cues include blushing, fidgeting, turning away, looking down at the floor, and a marked change in the breathing pattern.

Specific recommended screening questions about consumption include the following: About how many days per week do you drink alcohol? On a day when you do drink, how much do you drink? How many days per month do you have five or more drinks? These are often the first questions family physicians ask patients they suspect as having alcohol-related problems.

It may be useful to follow consumption questions with the CAGE questions (Ewing 1984) (acronym for “Have you ever felt you should Cut down on your drinking?” “Have people Annoyed you by criticizing your drinking?” “Have you ever felt bad or Guilty about your drinking?” “Have you ever had a drink first thing in the morning to steady your nerves or to get rid of a hangover [Eye opener]?”). Further queries about prior consequences of drinking are then useful. (For more information on screening instruments that may be helpful to family physicians, see Nilssen and Cone, pp. 136–139.)

Once a patient has screened positive for at-risk drinking, a family physician should assess that patient for evidence of physical dependence; the presence of serious alcohol-related health problems; use of illicit drugs; and social, legal, family, and employment problems. This information will facilitate the next step and assist the family physician in developing a treatment plan for the patient.

## Brief Interventions: The First Stage in Treatment

In contrast to the traditional opinion that long-term counseling for alcohol abuse is always necessary, there is increasing evidence that brief intervention, delivered by a physician, can reduce effectively alcohol use by persons at risk for alcohol-related problems. Several studies conducted in European and other countries in the 1980’s demonstrated 10 to 20 percent reductions in alcohol use in members of experimental groups who were given advice by general practitioners, compared with members of control groups who received no guidance (Kristenson et al. 1983; Saunders et al. 1993). These findings are consistent in more than 10 trials (Saunders et al. 1993). Effectiveness rates for brief intervention methods are similar to those of traditional alcoholism treatment programs with at-risk drinkers.

In a brief intervention, the physician offers a clear message to a patient about reducing alcohol consumption, often delivered in the context of a health, social, or family problem or concern (e.g., “I’m concerned about your stomach pain, and I think your alcohol use may be a part of the problem.”). Based on clinical judgment regarding the seriousness of the presenting problem, the family practitioner will determine if the patient’s problem warrants abstinence or a reduced level of drinking. To accomplish this, the physician might say, “I want you to stop drinking any alcohol for the next month so we can see if your stomach pain decreases” (if the patient cannot abstain despite medical problems, this indicates a more serious alcohol problem requiring follow-up); or “I want you to reduce your alcohol use to no more than one drink every other day. How do you feel about the amount?” (this provides an opportunity for some minor negotiation about the amount and can be completed in a structured 15-minute office visit).

Brief intervention also may involve giving the patient a pamphlet or self-help booklet, conducting followup telephone calls (usually 2 weeks after an office visit to determine if the patient is able to keep to the agreed-upon alcohol limit and to handle any problems that have arisen), and seeing the patient for followup visits to support behavior changes (usually at 1 month intervals).

## Specialized Treatment and Referral Methods

Patients who require specialized treatment have an increasing number of options. Alcoholics Anonymous and other 12-step models are still the most common foundation for treatment and long-term recovery. However, within the last 10 years, the alcohol field has moved away from the standard 28-day inpatient program, often called the Minnesota model, to several other models. These include not only traditional AA-based day and evening programs but also client-centered treatment that uses cue therapy, cognitive behavioral therapy, and motivation enhancement approaches. These approaches often use workbooks and other methods that include reasons to cut down or quit drinking, guides to risky situations, ways to cope with these situations, and ways to maintain treatment momentum.

Trends in treatment reflect a greater freedom of choice in treatment options. Providers are becoming increasingly oriented toward individualizing treatment for patients depending on their motivation to change, the presence of psychiatric and medical problems, and any family issues.

Patients who display physical dependence, severe alcohol-related health problems, and severe social disorders or are unable to change drinking behavior should be referred to an alcohol and other drug problems treatment specialist by their family physician. The physician can use several methods to identify such specialists in the community:

- Ask colleagues for names of treatment programs or individual providers.
- Contact an alcohol problems treatment specialist or program, mental health center, and/or hospital for consultation about a patient’s problem.
- Call the State alcohol and drug abuse agency for a list of the publicly and privately funded treatment programs in the State.
- Consult employee assistance programs in the area.
- Complete the Alcoholism Treatment Resources Guide that lists the telephone numbers of key professionals in the community (table 2). This resources guide could be posted in the nursing station, the reception area, or an examining room.

When referring a patient to a specialist, the family physician should:

- Advise the patient that a second opinion from a specialist should be obtained.
- Make telephone calls to alcohol problems specialists while the patient is in the examining room or have the patient make the appointment before he or she leaves the office.
- Ask specialists to call once they have evaluated patients. This allows family practitioners to participate in the treatment planning and support long-term behavioral change.

If patients refuse to see an alcohol problems treatment specialist or do not have financial resources, the family physician can:

- Identify recovering alcoholics in the physician’s own practice who are willing to meet with at-risk drinkers and alcoholics to discuss methods they can use to change their drinking behavior.
- Ask patients to attend mutual-help group meetings such as AA. Patients should be advised that they may have to attend several meetings in different locations to find compatible groups that fit their needs. Group meetings that do not use a 12-step approach include Rational Recovery and Women for Sobriety.

## Future Role for Family Physicians

Family physicians may assume an increasingly important role in the care of their patients with alcohol problems (Skinner 1990; Prochaska and DiClemente 1992) and can make a difference in several areas. They can:

- Establish effective screening procedures to identify at-risk drinkers and those who meet criteria for dependence.
- Conduct brief interventions with patients who meet the cutoff criteria for at-risk drinking.
- Ask an alcohol and other drug problems treatment specialist to conduct assessments and counseling in the primary care office.
- Identify affected members of families and provide treatment and referral.
- Use pharmacotherapy for craving, drug maintenance, and detoxification.
- Appropriately treat the recovering patient’s medical, surgical, or other needs to avoid prescribing mood-altering and addictive drugs.

Models for screening, assessing, and advising patients regarding alcohol use are available to assist family physicians in the management of one of the most prominent health care problems in the United States. As evidence supporting the effectiveness of screening and brief interventions causes trends in the treatment of alcohol-related problems to shift, the role of family physicians in the care of their patients with alcohol problems may be broadened.

## Figures and Tables

**Table 1:** Common Signs and Symptoms of Alcohol Problems Seen in Primary Care Settings

Medical Symptoms
Stomach/abdominal pain
Elevated blood pressure
Chronic tension headaches
Insomnia with associated medication request
Sexually transmitted diseases
Frequent accidents and trauma
Fatigue
Chronic depression
Chronic diarrhea
Memory loss

**Family Signs**

Frequent visits by other family members
Unexplained medical symptoms, such as headaches or abdominal pain in a child
Trauma in family members secondary to physical abuse
School problems in a child
Attention deficit disorder in a child
Depression or anxiety disorders in a child or spouse/partner

**Table 2:** Alcoholism Treatment Resources Guide

**1.**	**Alcohol and other drug problems treatment specialist**					
	Name _____________________________________________________________________________________					
	Phone _____________________________________________________________________________________					
	Name _____________________________________________________________________________________					
	Phone _____________________________________________________________________________________					
**2.**	**Physician with expertise in alcohol and other drug disorders**					
	Name _____________________________________________________________________________________					
	Phone _____________________________________________________________________________________					
**3.**	**State resource (RADAR Network Center**^1^**) phone number** ___________________					
**4.**	**AA phone numbers** ____________________________________________________________________					
**5.**	**Community substance abuse services (publicly funded)**					
	Name _____________________________________________________________________________________					
	Phone _____________________________________________________________________________________					
	Contact person ___________________________________________________________________________					
	Type of facility (circle):	residential	adolescent	outpatient	adult	evening
	Specialized program _____________________________________________________________________					
**6.**	**Veterans Affairs treatment resources**					
	Name _____________________________________________________________________________________					
	Phone _____________________________________________________________________________________					
	Hours _____________________________________________________________________________________					
	Contact person ___________________________________________________________________________					
	Type of facility (circle):	residential	adolescent	outpatient	adult	evening
	Specialized program _____________________________________________________________________					
**7.**	**Other treatment programs**					
	Name _____________________________________________________________________________________					
	Phone _____________________________________________________________________________________					
	Hours _____________________________________________________________________________________					
	Contact person ___________________________________________________________________________					
	Type of facility (circle):	residential	adolescent	outpatient	adult	evening
	Specialized program _____________________________________________________________________					
	Name _____________________________________________________________________________________					
	Phone _____________________________________________________________________________________					
	Hours _____________________________________________________________________________________					
	Contact person ___________________________________________________________________________					
	Type of facility (circle):	residential	adolescent	outpatient	adult	evening
	Specialized program _____________________________________________________________________					

1 The Regional Alcohol and Drug Awareness Resource (RADAR) Network is sponsored by the Federal Center for Substance Abuse Prevention through its National Clearinghouse for Alcohol and Drug Information. The RADAR Network offers its members priority access to information, publications, funding sources, and audiovisual materials developed by Federal agencies, other Network members, and national organizations.
